# Context encoding enables machine learning-based quantitative photoacoustics

**DOI:** 10.1117/1.JBO.23.5.056008

**Published:** 2018-05-18

**Authors:** Thomas Kirchner, Janek Gröhl, Lena Maier-Hein

**Affiliations:** a German Cancer Research Center (DKFZ), Division of Computer Assisted Medical Interventions (CAMI), Heidelberg, Germany; b Heidelberg University, Faculty of Physics and Astronomy, Heidelberg, Germany; c Heidelberg University, Medical Faculty, Heidelberg, Germany

**Keywords:** photoacoustics, quantification, multispectral imaging, machine learning

## Abstract

Real-time monitoring of functional tissue parameters, such as local blood oxygenation, based on optical imaging could provide groundbreaking advances in the diagnosis and interventional therapy of various diseases. Although photoacoustic (PA) imaging is a modality with great potential to measure optical absorption deep inside tissue, quantification of the measurements remains a major challenge. We introduce the first machine learning-based approach to quantitative PA imaging (qPAI), which relies on learning the fluence in a voxel to deduce the corresponding optical absorption. The method encodes relevant information of the measured signal and the characteristics of the imaging system in voxel-based feature vectors, which allow the generation of thousands of training samples from a single simulated PA image. Comprehensive *in silico* experiments suggest that context encoding-qPAI enables highly accurate and robust quantification of the local fluence and thereby the optical absorption from PA images.

## 1 Introduction

Photoacoustic (PA) imaging is an imaging concept with a high potential for real-time monitoring of functional tissue parameters such as blood oxygenation deep inside tissue. It measures the acoustic waves arising from the stress-confined thermal response of optical absorption in tissue.^1^ More specifically, a PA signal S(v) in a location v is a pressure response to the locally absorbed energy H(v), which, in turn, is a product of the absorption coefficient $\mu_{a}(v)$, the Grueneisen coefficient Γ(v) and the light fluence ϕ(v)

$$S(v)\propto H(v)=\mu_{a}(v)\cdot \Gamma (v)\cdot \phi (v).$$ (1)

Given that the local light fluence not only depends on the imaging setup but is also highly dependent on the optical properties of the surrounding tissue, quantification of optical absorption based on the measured PA signal is a major challenge.^2^^,^^3^ So far, the field of quantitative PA imaging (qPAI) has focused on model-based iterative optimization approaches to infer optical tissue parameters from measured signals (cf. e.g., Refs. 3–12). Although these methods are well suited for tomographic devices with high image quality (cf. e.g., Refs. 13–15) as used in small animal imaging, translational PA research with clinical ultrasound transducers or similar handheld devices (cf. e.g., Refs. 1 and 16–22) focuses on qualitative image analysis.

As an initial step toward clinical qPAI, we introduce a machine learning-based approach to quantifying PA measurements. The approach features high robustness to noise while being computationally efficient. In contrast to all other approaches proposed to date, our method relies on learning the light fluence on a voxel level to deduce the corresponding optical absorption. Our core contribution is the development of a voxel-based context image (CI) that encodes relevant information of the measured signal voxel together with characteristics of the imaging system in a single feature vector. This enables us to tackle the challenge of fluence estimation as a machine learning problem that we can solve in a fast and robust manner. Comprehensive *in silico* experiments indicate high accuracy, speed, and robustness of the proposed context encoding (CE)-qPAI approach. This is demonstrated for estimation of (1) fluence and optical absorption from PA images, as well as (2) blood oxygen saturation as an example of functional imaging using multispectral PA images.

## 2 Materials and Methods

A common challenge when applying machine learning methods to biomedical imaging problems is the lack of labeled training data. In the context of PAI, a major issue is the strong dependence of the signal on the surrounding tissue. This renders separation of voxels from their context—as in surface optical imaging^23^—impossible or highly inaccurate. Simulation of a sufficient number of training volumes covering a large range of tissue parameter variations, on the other hand, is computationally not feasible given the generally long runtime of Monte Carlo methods, which are currently the gold standard for the simulation of light transportation in tissue.^11^

**Fig. 1:**
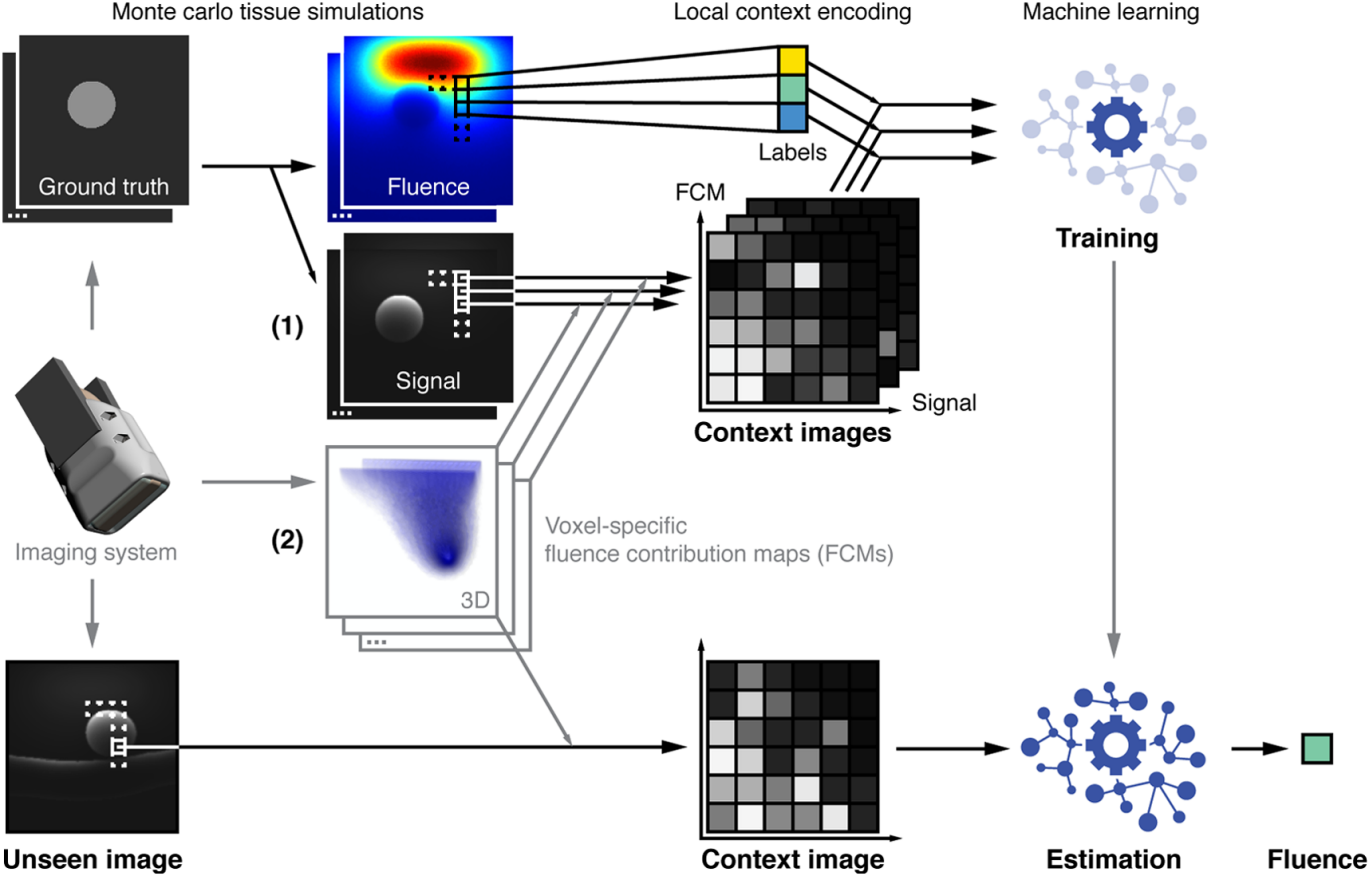
Machine learning approach to fluence estimation with CIs. CIs are generated individually for each voxel and encode both (1) relevant information on the measured signal extracted from the PAI signal volume and (2) prior knowledge on the characteristics of the imaging system represented by FCMs. During algorithm training, a regressor is presented tuples of CIs and corresponding ground truth fluence values for each voxel in the training data. For estimation of optical absorption in voxels of a previously unseen image, the voxel-specific CI is generated and used to infer the local fluence using the trained regressor.

Inspired by an approach to shape matching, where the shape context is encoded in a so-called spin image specifically for each node in a mesh,^24^ we encode the voxel-specific context in so-called CIs. This allows us to train machine learning algorithms on a voxel level rather than image level and we thus require orders of magnitude fewer simulated training volumes. CIs encode relevant information of the measured signal as well as characteristics of the imaging system represented by so-called voxel-specific fluence contribution maps (FCMs). The CIs serve as a feature vector for said machine learning algorithm, which is trained to estimate fluence in a voxel. The entire quantification method is shown in Fig. 1, which serves as an overview with details given in the following sections.

### 2.1 Fluence Contribution Map

**Fig. 2:**
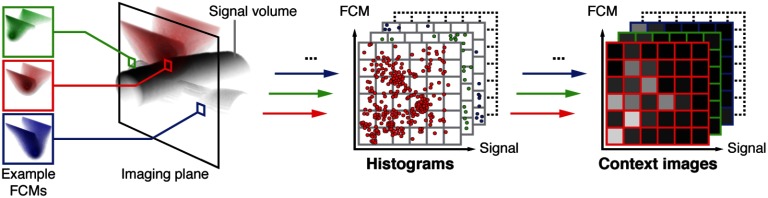
Generation of CIs for three representative voxels based on their FCMs. The voxel-specific FCMs serve as a representation of how the PA excitation light pulse propagates through homogeneous tissue to arrive in a target voxel given a chosen hardware setup. For each voxel (here: green, red, and blue), tupels of measured signal and corresponding fluence contribution (for that voxel) are determined to generate the voxel-specific histograms from which the CI is generated.

An important prerequisite for computing the CI for a voxel v is the computation of the corresponding FCM, referred to as FCM[v]. $\mathrm{FCM}[v](v^{\prime })$ represents a measure for the likelihood that a photon arriving in voxel v has passed $v^{\prime }$. In other words, an FCM reflects the impact of a PA signal in $v^{\prime }$ on the drop in fluence in voxel v. An illustration of an FCM corresponding to a typical handheld PA setup is shown in Fig. 2. The FCM[v] is dependent on how the PA excitation light pulse propagates through homogeneous tissue to arrive in v given a chosen hardware setup. The x×y FCMs per imaging plane are generated once for each new hardware setup and each voxel in the imaging plane.

In this first implementation of the CE-qPAI concept, FCMs are simulated with the same resolution as the input data assuming a background absorption coefficient of $0.1\;{\mathrm{cm}}^{-1}$ and a constant reduced scattering coefficient of $1.5\;{\mathrm{cm}}^{-1}$.^25^ The number of photons is varied to achieve a consistent photon count in the target voxel. The FCMs are generated with the widely used Monte Carlo simulation tool mcxyz.^26^ We integrated mcxyz into the open-source Medical Image Interaction Toolkit MITK^27^ as mitkMcxyz and modified it to work in a multithreaded environment. Sample FCMs for three different voxels are shown in Fig. 2, which also shows the generation of CIs for those three example voxels.

### 2.2 Context Image

The CI for a voxel v in a PA volume is essentially a two-dimensional (2-D) histogram composed of (1) the measured PA signal S in the tissue surrounding v and (2) the corresponding FCM[v]. More specifically, it is constructed from the tuples $\left\{(S(v^{\prime }),\mathrm{FCM}[v](v^{\prime }))|v^{\prime }\in N(v)\right\}$ where N(v) is defined as $N(v)=\{v^{\prime }|\mathrm{FCM}[v](v^{\prime })>\epsilon \}$. This constraint is set to exclude voxels with a negligible contribution to the fluence in v. The tuples are arranged by magnitude of $S(v^{\prime })$ and $\mathrm{FCM}[v](v^{\prime })$ into a 2-D histogram and thereby encode the relevant context information in a compact form. In our prototype implementation of the CE-qPAI concept, the fluence contribution and signal axes of the histogram are discretized in 12 bins and scaled logarithmically to better represent the predominantly low signal and fluence contribution components. The ranges of the axes are set as 0<log(S)<log(255) and log(ϵ)<log(FCM)<−1. Signals and fluence contributions larger than the upper boundary are included in the highest bin, whereas smaller signals and fluence contributions are not. Figure 2 shows the generation of CIs from FCMs and PA signals. Labeled CIs are used for training a regressor that can later estimate fluence, which, in turn, is used to reconstruct absorption [Eq. (1)].

### 2.3 Machine Learning-Based Regression for Fluence Estimation

During the training phase, a regressor is presented tuples [CI(v),ϕ(v)] of CI(v) and corresponding ground truth fluence values ϕ(v) for each voxel v in a set of PAI volumes. For estimation of optical absorption in a voxel $v_{u}$ of a previously unseen image, the voxel-specific CI is generated and used to infer fluence ϕ^(vu) using the trained algorithm.

In our prototype implementation of the CE-qPAI method, we use a random forest regressor. A random forest regressor is an ensemble of decision trees, where the weighted vote of the individual trees is used as the estimation.^28^ To train the random forest, all labeled CIs of the respective training set need to be evaluated at once. With voxel-based CIs, thousands of training samples can be extracted from a single slice of a simulated PA training volume. Ground truth training data generation is performed using a dedicated software plugin integrated into MITK and simulating the fluence with mitkMcxyz. It should be noted that the simulated images consist mainly of background voxels and not of vessel structures, which are our regions of interest (ROI). This leads to an imbalance in the training set. To avoid poor estimation for underrepresented classes,^29^ we undersample background voxels in the training process to ensure a 1:1 ROI/background sample ratio. The parameters of the random forest are set to the defaults of sklearn 0.18 using python 2.7, except for the tree count which was set to $n_{\text{regressors}}\;=100$. CIs are used as feature vectors and labeled with the optical property to be estimated (e.g., fluence or oxygenation). The parameters were chosen based on a grid search on a separate dataset not used in the experiments of this work.

### 2.4 Hardware Setup

We assume a typical linear probe hardware setup,^30^ where the ultrasound detector array and the light source move together and the illumination geometry is the same for each image recorded. This is also the case for other typical tomographic devices.^31^^,^^32^ All simulations were performed on high-end CPUs (Intel i7-5960X).

## 3 Experiments and Results

In the following validation experiments, we quantify the fluence up to an imaging depth of 28 mm in unseen test images for each dataset. With our implementation and setup, all images comprise 3008 training samples, which results in an average simulation time of about 50 ms per training sample. This allows us to generate enough training samples in a feasible amount of time, to train a regressor that enables fluence estimation in a previously unseen image in near real time. The measured computational time for quantifying fluence in a single 64×47  voxel image slice is 0.9  s±0.1  s.

In the following, we present the experimental design and results of the validation of CE-qPAI. First, we will validate the estimation of absorption from PAI volumes acquired at a fixed wavelength and then estimate blood oxygenation from multispectral PAI volumes.

### 3.1 Monospectral Absorption Estimation

#### 3.1.1 Experiment

To assess the performance of CE-qPAI in PA images of blood vessels, we designed six experimental datasets (DS) with varying complexities as listed in Table 1. With the exception of ${\mathrm{DS}}_{\mathrm{multi}}$, each of the six experimental DS is composed of 150 training items, 25 validation items, and 25 test items, where each item comprises a three-dimensional (3-D) simulated PA image of dimensions 64×47×62 and 0.6-mm equal spacing as well as a corresponding (ground truth) fluence map.

**Table 1:** The design parameters of the DS. All ranges denote sampling from uniform distributions within the given bounds.

Dataset	Vessel radius [mm]	Vessel absorption $\mu_{a}$ [${\mathrm{cm}}^{-1}$]	Vessel count	Background absorption $\mu_{a}$ [${\mathrm{cm}}^{-1}$]
${\mathrm{DS}}_{\text{base}}$	3	4.7	1	0.1
${\mathrm{DS}}_{\text{radius}}$	**0.5** to **6**	4.7	1	0.1
${\mathrm{DS}}_{\mathrm{absorb}}$	3	**1** to **12**	1	0.1
${\mathrm{DS}}_{\text{vessel}}$	3	4.7	**1** to **7**	0.1
${\mathrm{DS}}_{\text{background}}$	3	4.7	1	$10^{-4}$ to **0.2**
${\mathrm{DS}}_{\mathrm{multi}}$	0.5 to 6	1 to 12	1 to 7	$10^{-4}$ to 0.2

As labels of the generated CIs, we used a fluence correction $\phi_{c}(v^{\prime })=\phi (v^{\prime })/\phi_{h}(v^{\prime })$, where $\phi_{h}(v^{\prime })$ is a fluence simulation based on a homogeneous background tissue assumption. We used five equidistant slices out of each volume, resulting in a generation of a total of 2,256,000; 376,000 and 376,000 CIs for each dataset—for training, parameter optimization, and testing, respectively. To account for the high complexity of ${\mathrm{DS}}_{\mathrm{multi}}$, we increased the number of training volumes for that set from 150 to 400. The baseline dataset ${\mathrm{DS}}_{\text{base}}$ represents simulations of a transcutaneously scanned simplified model of a blood vessel of constant radius (3 mm) and constant absorption (vessel: $4.73\;{\mathrm{cm}}^{-1}$, background: $0.1\;{\mathrm{cm}}^{-1}$) and reduced scattering coefficient ($1.5\;{\mathrm{cm}}^{-1}$). To approximate partial volume effects, the absorption coefficients in the ground truth images were Gaussian blurred with a sigma of 0.6 mm. Single slices were simulated using $2\times 10^{6}$ photons for all training sets and $10^{8}$ photons for the respective test and validation sets and then compounded in a fully scanned volume. Different shapes and poses of the vessel were generated by a random walk with steps r defined as

$$r_{i}=r_{i-1}+\eta \cdot a,$$ (2)

where η is a free parameter constant in each vessel with an inter-vessel variation within a uniform distribution (0<η<0.2) and a is varied for each of its components in each step within a uniform distribution $\left(-0.2\;\mathrm{mm}<a_{i}<0.2\;\mathrm{mm}\right)$. To investigate how variations in geometry and optical properties impact the performance of our method, we designed further experimental DS in which the number of vessels (${\mathrm{DS}}_{\text{vessel}}$), the radii of the vessels (${\mathrm{DS}}_{\text{radius}}$), the optical absorption coefficients within the vessels (${\mathrm{DS}}_{\mathrm{absorb}}$), the absorption coefficient of the background (${\mathrm{DS}}_{\text{background}}$), as well as all of the above (${\mathrm{DS}}_{\mathrm{multi}}$) were varied. We tested the robustness of CE-qPAI to this range of scenarios without retuning CI or random forest parameters.

Although most studies assess the performance of a method in the entire image (cf. e.g., Refs. 6, 33, and 34), it must be pointed out that the accuracy of signal quantification is often most relevant in a defined region of interest—such as in vessels or regions that provide a meaningful PA signal. These are typically also the regions, where quantification is particularly challenging due to the strongest signals originating from boundaries with discontinuous tissue properties. To address this important aspect we validated our method, not only on the entire image, but also in the ROI, which we define for our DS as voxels representing a vessel and at the same time having a contrast-to-noise ratio (CNR) of larger than 2, to only include significant signal in the ROI. We define CNR following Walvaert and Rosseel^35^ in a voxel v as

$$\mathrm{CNR}=\frac{S(v)-\mathrm{avg}(b)}{\mathrm{std}(b)},$$ (3)

where the avg(b) and std(b) are the average and standard deviations of the background signal b over a simulated image slice with a background absorption coefficient of $0.1\;{\mathrm{cm}}^{-1}$ and no other structures. Using such an image without application of a noise model, we simulated an intrinsic background noise of (4.2±2.8) a.u.

To investigate the robustness of CE-qPAI to noise, we added the following noise models to each dataset. The noise models consist of an additive Gaussian noise term applied on the signal volumes followed by a multiplicative white Gaussian noise term, similar to noise assumptions used in prior work.^6^^,^^33^ We examined three noise levels to compare against the simulation-intrinsic noise case:

1. 2% multiplicative and (0.125±0.125) a.u. additive component
2. 10% multiplicative and (0.625±0.625) a.u. additive component
3. 20% multiplicative and (1.25±1.25) a.u. additive component

The additive and multiplicative noise components follow an estimation of noise components on a custom PA system.^30^ For each experimental dataset introduced in Table 1 and each noise set, we applied the following validation procedure separately. Following common research practice, we used the training data subset for training of the random forest and the validation data subset to ensure the convergence of the training process, as well as to set suitable parameters for the random forest and ROI, whereas we only evaluated the test data subset to report the final results (as described in Ref. 36). As an error metric, we report the relative fluence estimation error $e_{r}$

er(v)=|ϕ^(v)−ϕ(v)|ϕ(v), (4)

rather than an absorption estimation error, to separate the error in estimating fluence with CE-qPAI from errors introduced through simulation-intrinsic or added noise on the signal, which will affect the quantification regardless of fluence estimation.

#### 3.1.2 Results

**Fig. 3:**
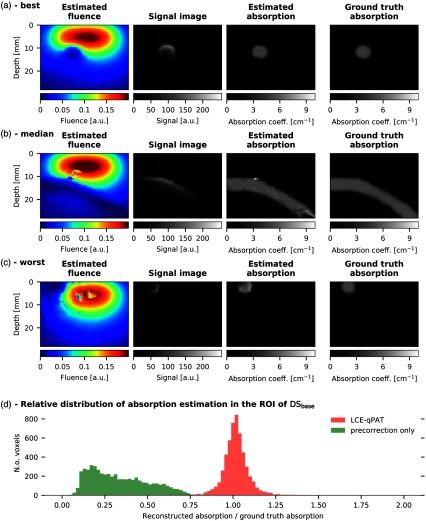
Absorption reconstruction results after fluence estimation. For the slices with the (a) lowest, (b) median, and (c) highest median fluence estimation error $e_{r}$ within the ROI of ${\mathrm{DS}}_{\text{base}}$. We show (from left to right) the estimated fluence, the corresponding signal images, the resulting estimation of the absorption coefficient, and the ground truth optical absorption, for reference. (d) A histogram of the relative absorption estimation over all ROI voxels (n=5347) in ${\mathrm{DS}}_{\text{base}}$ illustrating absorption estimation accuracy rather than fluence estimation accuracy measured by $e_{r}$. Precorrecting the signal with the fluence of a homogeneous tissue assumption underestimates the absorption and is considerably outperformed by CE-qPAI in the ROI. The CE-qPAI plot omits 5 outliers larger 2.

Figures 3(a)–3(c) show representative examples of the previously unseen 125 simulated test images from the baseline dataset ${\mathrm{DS}}_{\text{base}}$, with their corresponding fluence estimation results. The optical absorption is reconstructed using the fluence estimation. A histogram illustrating absorption estimation accuracy in ROI voxels of ${\mathrm{DS}}_{\text{base}}$ is shown in Fig. 3(d) and compared with a static fluence correction approach.

Table 2 summarizes the descriptive statistics of the relative fluence estimation errors $e_{r}$ for the experiments on absorption estimation using single wavelength PA images. The relative fluence estimation error $e_{r}$ does not follow a normal distribution due to large outliers especially in complex DS, which is why we report median $e_{r}$ with interquartile ranges (IQR) for all DS. Even for the most complex dataset ${\mathrm{DS}}_{\mathrm{multi}}$ with variations of multiple parameters, CE-qPAI yields a median overall relative fluence estimation error $e_{r}$ below 4%. Errors are higher in the ROI, especially in DS with high variations of absorption.

**Table 2:** Descriptive statistics of fluence estimation results. The median and IQR of the relative fluence estimation error $e_{r}$ for the six validation DS used for the single wavelength experiments. The median error and IQR are provided (1) for all voxels in the respective test set as well as (2) for the voxels in the ROI only.

Relative error $e_{r}$				
	All voxels		ROI	
Dataset	Median (%)	IQR (%)	Median (%)	IQR (%)
${\mathrm{DS}}_{\text{base}}$	1.0	(0.5, 1.9)	4.2	(1.9, 7.6)
${\mathrm{DS}}_{\text{radius}}$	1.4	(0.6, 3.3)	5.7	(2.4, 11.3)
${\mathrm{DS}}_{\mathrm{absorb}}$	1.2	(0.5, 2.8)	14.7	(5.4, 32.2)
${\mathrm{DS}}_{\text{vessel}}$	1.8	(0.7, 6.2)	6.8	(3.0, 13.2)
${\mathrm{DS}}_{\text{background}}$	0.7	(0.3, 1.4)	4.1	(1.7, 7.3)
${\mathrm{DS}}_{\mathrm{multi}}$	2.3	(0.7, 38.5)	15.7	(6.6, 40.0)

**Fig. 4:**
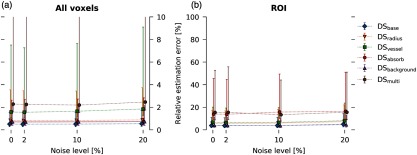
Robustness of the fluence estimation against noise. Median relative fluence estimation errors $e_{r}$ with IQR over all DS for, (a) all test voxels, and (b) in region of interest test voxels. The whiskers in this plot show the first and third quartile.

Previously proposed qPAI approaches reveal high drops in estimation performance when dealing with noisy data (cf. e.g., Ref. 37). To remedy this, methods have been proposed to incorporate more accurate noise representations into model-based reconstruction algorithms.^33^^,^^38^ When validating the robustness of CE-qPAI to noise, it yields high accuracy even under unrealistically high noise levels of up to 20% (cf. Fig. 4). Regardless of the noise level applied, the highest median errors occur in the ROIs of DS that are characterized by high absorption and inhomogeneous tissue properties.

### 3.2 Multispectral Blood Oxygenation Estimation

The concept of CE cannot only be used to estimate fluence and absorption, but also derived functional parameters such as blood oxygenation. To this end, the estimated absorption in a voxel for multiple wavelengths can be applied to resolve oxygenation via linear spectral unmixing. Alternatively, a regressor can be trained using the CIs labeled with ground truth oxygenation.

#### 3.2.1 Experiment

To investigate the performance of CE-qPAI for blood oxygenation (${\mathrm{sO}}_{2}$) estimation, we designed an additional multispectral simulated dataset ${\mathrm{DS}}_{\mathrm{oxy}}$ using the wavelengths 750, 800, and 850 nm. It consists of 240 multispectral training volumes and 11 multispectral test volumes, each featuring homogeneous oxygenation and one vessel with a radius of 2.3 to 4 mm—modeled after a carotid artery.^39^ For each image slice and at each wavelength, $10^{7}$ photons were used for simulation. Oxygenation values for the training images were drawn randomly from a uniform ${\mathrm{sO}}_{2}$ distribution U(0%,100%). For testing, we simulated 11 multispectral volumes at three wavelengths and 11 blood oxygenation levels (${\mathrm{sO}}_{2}\in \{0\%,10\%,20\%,\ldots ,100\%\}$). The optical absorption was adjusted by wavelength and oxygenation, as described by Jacques.^25^ Hemoglobin concentration was assumed to be 150  g/L.^25^ The blood volume fraction was set to 0.5% in the background tissue and to 100% in the blood vessels. The reduced scattering coefficient was again set to $1.5\;{\mathrm{cm}}^{-1}$. We estimated the oxygenation using three methods:

1. *Linear spectral unmixing on the signal images as a baseline*.^40^ For this, we applied a non-negative constrained least squares approach as also used in Ref. 15 that minimizes ‖Ax−b‖=0, where A is the matrix containing the reference spectra, b is the measurement vector, and x is the unmixing result. Specifically, we used the python scipy.optimize.minimize function with the sequential least squares programming method and added a non-negativity inequality constraint. We evaluated the unmixing results of this method on all voxels in the ROI as well as exclusively on those voxels with the maximum intensity projection (MIP) along image x-axis at wavelength 800 nm to account for nonlinear fluence effects deep inside the vessels.
2. *Linear spectral unmixing of the signal after quantification of the three input images with CE-qPAI.* After correcting the raw signal images for nonlinear fluence effects using CE-qPAI, we applied the same method as described in (1) and evaluated on the same voxels that were used in (1) to ensure comparability of the results.
3. *Direct estimation of oxygenation using a functional adaptation of CE-qPAI.* For functional CE-qPAI (fCE-qPAI), triples of CIs for the three chosen wavelengths were concatenated into one feature vector and labeled with the ground truth oxygenation.

#### 3.2.2 Results

**Fig. 5:**
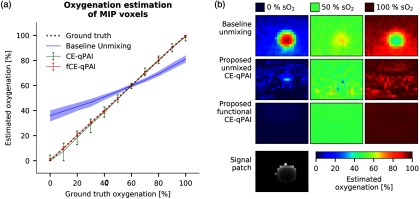
Oxygenation estimation. (a) The median oxygen estimation with the IQR on the MIP voxels using linear spectral unmixing of (blue) the uncorrected signal, (green) the signal corrected by CE-qPAI, and (red) direct estimation by functional CE-qPAI (fCE-qPAI). (b) The oxygenation estimation for a representative patch of signal showing a vessel in 15-mm depth and with 3-mm radius. The signal for one of the measurement wavelengths is shown for reference together with the oxygen estimation results for 0%, 50%, and 100% ground truth homogeneous oxygenation and the three examined methods.

Estimation of local blood oxygen saturation (${\mathrm{sO}}_{2}$) is one of the main qPAI applications and is only possible with multispectral measurements. As such, the presented approaches were validated together with the baseline method on the dataset ${\mathrm{DS}}_{\mathrm{oxy}}$. As shown in Fig. 5(a), the estimation results for both methods are in very close agreement with the ground truth. In fact, the median absolute oxygen estimation error was 3.1% with IQR (1.1% and 6.4%) for CE-qPAI and 0.8% with IQR (0.3% and 1.8%) for the fCE-qPAI adaptation. Furthermore, our methodology outperforms a baseline approach based on linear spectral unmixing of the raw signal (as also compared to in Ref. 15). By means of example Fig. 5(b) shows that the linear spectral unmixing of the ROI on the uncorrected signal fails deep inside the ROI, where the fluence varies strongly for different wavelengths. To compensate for this effect when comparing the approach to our method, we validate all methods only on the MIP along the depth axis (as also used in Ref. 41) in Fig. 5(a).

## 4 Discussion

This paper addresses one of the most important challenges related to PA imaging, namely the quantification of optical absorption based on the measured signal. In contrast to all other approaches proposed to qPAI to date (cf. e.g., Refs. 3–12), our method relies on learning the light fluence in a voxel to deduce the corresponding optical absorption. Comprehensive *in silico* experiments presented in this manuscript show the high potential of this approach to estimate optical absorption as well as derived functional properties, such as oxygenation, even in the presence of high noise.

Although machine learning methods have recently been applied to PAI related problems (cf. e.g., Refs. 42–44), these have mainly focused on image reconstruction but not signal quantification. We attribute this to the fact that *in vivo* training data generation for machine learning-based qPAI is not at all straightforward given the lack of reference methods for estimating optical absorption in depth. Despite recent developments related to hybrid diffusion approximation and Monte Carlo methods,^45^ fast generation of *in silico* training data also remains an unsolved challenge. Note in this context that commonly applied methods of data augmentation (i.e., methods that may be used to automatically enlarge training data sets as discussed in Ref. 46) cannot be applied to PA images due to the interdependence of fluence and signal. With our contribution, we have addressed the challenge by introducing the concept of CIs, which allow us to generate one training case from each voxel rather than from each image.

As an important contribution with high potential impact, we adapted CE-qPAI to estimate functional tissue properties from multiwavelength data. Both variants—linear spectral unmixing of the fluence corrected signal, as well as direct estimation of oxygenation from multi wavelength CIs, yielded accurate results that outperformed a baseline approach based on linear spectral unmixing of the raw PA signal. It should be noted that linear spectral unmixing of the signal for ${\mathrm{sO}}_{2}$ estimation is usually performed on a wider range of wavelengths to increase accuracy. However, even this increase in the number of wavelengths cannot fully account for nonlinear fluence effects.^3^ Combined with the separately established robustness to noise, multiwavelength applications of CE-qPAI are very promising.

In our first prototype implementation of CE-qPAI, we used random forests regressors with standard parameters. It should be noted, however, that fluence estimation from the proposed CI can in principle be performed by any other machine learning method in a straightforward manner. Initial experiments suggest that even better performance can be achieved with convolutional neural networks.^47^

By relating the measured signals $S(v^{\prime })$ in the neighborhood of v to the corresponding fluence contributions $\mathrm{FCM}[v](v^{\prime })$ we relate the absorbed energy in $v^{\prime }$, to the fluence contribution of $v^{\prime }$ to v. In this context, it has to be noted that the fluence contribution $\mathrm{FCM}[v](v^{\prime })$ is only an approximation of the true likelihood that a photon passing v has previously passed $v^{\prime }$, because FCM[v] is generated independently of the scene under observation assuming constant background absorption and scattering. Nevertheless due to the generally low variance of scattering in tissue, it serves as a reliable input for the proposed machine learning-based quantification.

A limitation of our study can be seen in the fact that we performed the validation *in silico*. To apply CE-qPAI *in vivo*, further research will have to be conducted in two main areas. First, we are working on accurately solving the acoustical inverse problem for specific scanners.^48^ The method will be integrated into the quantification algorithm to enable quantification of images acquired with common PAI probes such as clinical linear transducers. Second, training data have to be generated as close to reality as possible—considering, for example, imaging artifacts.

In contrast to prior work (cf. e.g., Refs. 6, 7, 33, 49, and 34), our initial validation handles the whole range of near infrared absorption in whole blood at physiological hemoglobin concentrations and demonstrates high robustness to noise. The impact of variations of scattering still needs investigation although these should be small in the near infrared.

Long-term goal of our work is the transfer of CE-qPAI to clinical data. In this context, run-time of the algorithm will play an important role. Although our current implementation can estimate absorption on single slices within a second, this might not be sufficient for interventional clinical estimation of whole tissue volumes and at higher resolutions. An efficient GPU implementation of the time intensive CI generation should enable real-time quantification.

In summary, CE-qPAI is the first machine learning-based approach to quantification of PA signals. The results of this work suggest that quantitative real-time functional PA imaging deep inside tissue is feasible.

## Code and Data Availability

The code for the method as well as the experiments was written in C++ and python 2.7 and is partially open source and available at https://phabricator.mitk.org/source/mitk.git. Additional code and all raw and processed data generated in this work are available from the corresponding authors on reasonable request.
