# Context encoding enables machine learning-based quantitative photoacoustics (Erratum)

**DOI:** 10.1117/1.JBO.25.4.049802

**Published:** 2020-04-16

**Authors:** Thomas Kirchner, Janek Gröhl, Lena Maier-Hein

**Affiliations:** a German Cancer Research Center (DKFZ), Division of Computer Assisted Medical Interventions (CAMI), Heidelberg, Germany; b Heidelberg University, Faculty of Physics and Astronomy, Heidelberg, Germany; c Heidelberg University, Medical Faculty, Heidelberg, Germany

## Abstract

The erratum corrects an error in the published article.

This article [*J. Biomed. Opt.* **23**(5), 056008 (2018) doi: 10.1117/1.JBO.23.5.056008 was originally published online on 18 May 2018 with an error in the reported number for the reduced scattering coefficient. The authors reported a reduced scattering coefficient of 15 cm^−1^; however, this number was not the reduced scattering coefficient, but the normal scattering coefficient.

To correct the error, the reported number for the reduced scattering coefficient has been adjusted from 15 cm^−1^ to 1.5 cm^−1^ in three instances in the article:

1. Section 2.1, Fluence Contribution Map, second paragraph;
2. Section 3.1.1, Experiment, second paragraph; and
3. Section 3.2.1, Experiment, first paragraph.

In the article, the authors claim the general feasibility of the proposed method towards fluence estimation from photoacoustic initial pressure data. By the opinion of all involved authors, this claim is not impeded by the reporting error.

This article was corrected online on 7 April 2020.

